# O055. Headache and psychopathological aspects in Gilles de la Tourette Sindrome: a comparison between paediatric and adult patients

**DOI:** 10.1186/1129-2377-16-S1-A140

**Published:** 2015-09-28

**Authors:** Valentina Bandera, Beatrice Bartoli, Chiara Luoni, Claudia Selvini, Giorgio Rossi, Umberto Balottin, Andrea Cavanna, Cristiano Termine

**Affiliations:** Child Neuropsychiatry Unit, Department of Clinical and Experimental Medicine, University of Insubria, Varese, Italy; Child and Adolescent Neuropsychiatry Unit, Ospedale del Ponte, Fondazione Macchi, Varese, Italy; Department of Child Neurology and Psychiatry, IRCCS “C. Mondino” Foundation, University of Pavia, Pavia, Italy; School of Life and Health Sciences, Aston University, Birmingham, UK; Department of Neuropsychiatry, BSMHFT and University of Birmingham, Birmingham, UK; Sobell Department of Motor Neuroscience and Movement Disorders, UCL and Institute of Neurology, London, UK

## Background

Only few studies have analyzed the occurrence of headache in patients with Gilles de la Tourette syndrome (GTS) [1–3]. The aim of this study was to compare the prevalence and characteristics of headache in paediatric and adult patients with GTS and the relationship of headache with tic severity, psychiatric comorbidities and quality of life.

## Materials and methods

One hundred and nine children and adolescents with GTS (age range, 6-17 years) were screened for the occurrence of headache between April and December 2014 and twenty-five GTS patients (23%) showed headache. Sixteen of these have been compared with eighteen randomly selected GTS patients without headache with reference to severity of tics, psychiatric comorbidities (OCD, ADHD, anxiety, depression) and quality of life, using specific rating scales and questionnaires. Thirty-one adult GTS patients, randomly recruited from a group of 200 patients, were screened for the presence of headache and underwent the same clinical assessment.

## Results

Adults with GTS, compared with children and adolescents, showed a higher prevalence of headache (48.4% vs 23%, p < 0.05), higher tic severity, lower quality of life and higher prevalence of associated comorbidities: OCD (77.4% vs 52.9%), anxiety (77.4% vs 32.4%), depression (64.5% vs 26.5%). Children and adolescent GTS patients with headache showed a lower severity and frequency of tics compared with GTS patients without headache.

## Conclusions

Adult patients with GTS show a higher prevalence of headache and a more severe clinical phenotype compared to younger patients. Among children and adolescents, those with headache showed a lower severity and frequency of tics, thus supporting the hypothesis that in young GTS patients, headache and tics could be considered different phenotypic expressions of a common etiopathogenetic mechanism (e.g., psychosomatic symptoms of poor anger and aggression management) [4, 5].

Written informed consent to publish was obtained from the patient(s).

## References

[1] Barabas G, Matthews WS, Ferrari M (1984). Tourette's Syndrome and migraine. Arch Neurol.

[2] Gosh D, Rajan PV, Das D, Datta P, Rothner AD, Erenberg G (2012). Headache in children with Tourette Syndrome. J Pediatr.

[3] Kwak C, Vuong KD, Jankovic J (2003). Migraine headache in patients with Tourette Syndrome. Am Med Ass.

[4] Cavanna AE, Selvini C, Luoni C, Eddy CM, Fizzah A, Blangiardo R (2014). Measuring anger expression in young patients with Tourette Syndrome. Children's Health Care.

[5] Lanzi G, Balottin U, Gamba N, Fazzi E (1983). Psychological aspects of migraine in childhood. Cephalalgia.

